# The cost of social influence: Own-gender and gender-stereotype social learning biases in adolescents and adults

**DOI:** 10.1371/journal.pone.0290122

**Published:** 2023-08-11

**Authors:** Sheila J. Cunningham, Jacqui Hutchison, Natalie Ellis, Ivana Hezelyova, Lara A. Wood

**Affiliations:** 1 Division of Psychology and Forensic Sciences, School of Applied Sciences Dundee, Dundee, United Kingdom; 2 School of Psychology, University of Aberdeen, Aberdeen, United Kingdom; Anhui Agricultural University, CHINA

## Abstract

Pervasive gender gaps in academic subject and career choices are likely to be underpinned by social influences, including gender stereotypes of competence in academic and career domains (e.g., men excel at engineering, women excel at care), and model-based social learning biases (i.e., selective copying of particular individuals). Here, we explore the influence of gender stereotypes on social learning decisions in adolescent and adult males and females. Participants (Exp 1: *N* = 69 adolescents; Exp 2: *N* = 265 adults) were presented with 16 difficult multiple-choice questions from stereotypically feminine (e.g., care) and masculine (e.g., engineering) domains. The answer choices included the correct response and three incorrect responses paired with a male model, a female model, or no model. Participants’ gender stereotype knowledge and endorsement were measured, and adolescents (Exp. 1) listed their academic subject choices. As predicted, there was a bias towards copying answers paired with a model (Exp.1: 74%, Exp. 2: 65% *p*s < .001). This resulted in less success than would be expected by chance (Exp. 1: 12%, Exp. 2: 16% *p*s < .001), demonstrating a negative consequence of social information. Adults (Exp 2) showed gender stereotyped social learning biases; they were more likely to copy a male model in masculine questions and a female model in feminine questions (*p* = .012). However, adolescents (Exp 1) showed no evidence of this stereotype bias; rather, there was a tendency for male adolescents to copy male models regardless of domain (*p* = .004). This own-gender bias was not apparent in female adolescents. In Exp 1, endorsement of masculine stereotypes was positively associated with selecting more own-gender typical academic subjects at school and copying significantly more male models in the male questions. The current study provides evidence for the first time that decision-making in both adolescence and adulthood is impacted by gender biases.

## Introduction

From infancy, humans acquire a wealth of information about the world through the social learning, the observation and copying of other people [1, 2]. Social learning is a form of social influence that can be hugely beneficial in terms of success within an environment but can also lead to the copying of inefficient or deleterious behaviour. Importantly, theoretical models and empirical evidence highlight that social learning is selective: not all that is observed is copied [3, 4]. Children and adults show biases in their decisions over what and whom to copy, influenced by factors such as the perceived competence of the model [5]. This competence perception may itself be biased by a second important social influence: stereotypic cultural associations, which lead to different social groups being perceived as competent at different tasks. Social learning choices may therefore be influenced by dominant stereotypes, such as a man being copied over a woman in an engineering task regardless of the models’ personal competence.

The potential for social learning and stereotypes to interact in this way is important because of their real-world impact on decision-making. For example, the social influences of both gender stereotypes and peer social learning have been implicated in the perpetuation of gender imbalances in academic subject and career choices, with long term economic and social status repercussions [see 6, 7]. In particular, caring professions like teaching and healthcare are female dominated while there is male domination in higher-status science, technology, engineering and maths (STEM) roles [8, 9]. Gender biases in social learning in adolescence and across adulthood may contribute to these gender imbalances.

Both young children and adults show a bias towards copying models with task-related expertise and competence [5, 10, 11], even when this social learning is not reinforced by feedback regarding the success of the model or observer [12, 13]. Copying also increases when the observer’s own confidence is low [14, 15]. When perceivers lack information about an individual model’s competency, social category information guides their selective copying. For example, unfamiliar models with no competency reputation are more likely to be copied in novel tasks if they are adults rather than children [16–18], with competence in the former being implied by their longer experience and assumed knowledge of their environment. This suggests that model group membership consciously or unconsciously guides copying choices, supporting the rationale that male and female models may be more likely to be copied within the fields of expertise stereotypically associated with their gender.

However, shared social group membership between the observer and an unfamiliar model can also bias model choice. For example, models are more likely to be trusted and copied if they share a language, or even accent, with the observer [19–21]. This preference for in-group copying has been shown to extend to gender categories in young children, who have a bias to copy the choices of same-sex models [22–25]. For example, Ma and Woolley presented 4- to 6-year-old children with male and female voices providing contradictory information about the function of novel objects, which were coloured pink, blue, or gender-neutral yellow [23, Study 1]. When asked what they believed the function of the objects to be, girls were more likely to copy the female model’s answer and boys were more likely to repeat the male model’s answer, regardless of the object’s colour and associated gender stereotype. Young children may therefore show an own-gender bias rather than cultural stereotype bias in their social learning choices, believing their own gender to be more competent regardless of the stereotypic competence association.

The early own-gender bias has been shown in studies across early to mid-childhood, with both boys and girls tending to believe their own gender to be most competent [26–28]. However, this belief is increasingly challenged by the steady acquisition of stereotypic associations [29, 30]. While five-year-old boys and girls both tend to believe their own gender is smarter, by six to seven years both genders associate academic brilliance with masculinity [26]. Similarly, in 4^th^ to 8^th^ grade children (aged approximately 8 to 14 years), Kurtz-Cortez and colleagues found a change in beliefs about verbal ability from own-gender bias in the younger children, to the cultural stereotypic that girls excel in this domain in the older participants [27]. The extent to which own-gender biases may prevail in adolescent social learning is therefore unclear. However, it seems likely that gender stereotypic competence beliefs may override own-gender competence beliefs by this stage, particularly for feminine domains.

Endorsement of gender competence stereotypes may also vary between males and females. Wood and colleagues developed a Gender Attribute (GA) scale to measure young people’s personal *endorsement* of gender stereotypes (e.g., “Who do you think would be better at science—boys, girls or both?”) as well as their *knowledge* of the cultural stereotype (e.g., “Who do most people think would be better at science—boys, girls or both?”, emphasis added) [7]. Responses showed high levels of stereotype knowledge in both genders, but there was also evidence of stereotype rejection: knowledge of cultural stereotypes was consistently higher than personal endorsement. This stereotype rejection was much higher in adolescent girls than boys. It is likely that girls will have been targeted more than boys by interventions encouraging rejection of gender stereotyped careers, which tend to focus on encouraging females into STEM subjects and careers [31]. Examination of the availability interventions to attract boys into stereotypically feminine domains, such as caring professions, suggests these are relatively uncommon despite current worker shortages in these areas [32]. Boys’ decision making may therefore be more affected by stereotypic barriers than girls’.

As well as a gender difference in stereotype attitudes, Wood and colleagues found a difference between boys’ and girls’ tendency to choose own-gender stereotypic academic subjects at school [7]. Boys’ choices comprised around 75% stereotypically masculine subjects on average, while females chose around 50% stereotypically associated with each gender. Further, in male participants only, stereotype rejection scores were negatively correlated with the proportion of gender-stereotype academic subjects chosen. In other words, boys (but not girls) who chose more own-gender subjects tended to show less stereotype rejection in their attitudes, highlighting the importance of understanding and addressing gender stereotype attitudes in teenage boys. This raises the possibility that boys will be particularly vulnerable to gender stereotype biases in their social learning.

It is important to note that while social learning is generally beneficial, biases towards copying social information can lead to the copying of functionally irrelevant [33], maladaptive [34], and even harmful behaviour [35]. Additionally, an important lesson from social cognition is that stereotypic biases in social processing lead to inaccuracies, due to exaggeration and overgeneralisation of group characteristics [36]. If perceivers inaccurately infer competence from social group membership and therefore copy an inaccurate model, this may lead to social learning diminishing rather than improving success. This aspect of social learning is an important consideration when applied to real world issues like academic and career choices. For example, when deciding which occupation to pursue, an adolescent boy may have an aptitude (e.g., *good at caring for people*) that is countered by both social learning (*males I know choose non-caring roles*) and gender stereotypes (*men are not good carers*). In this case, social influences may lead to the rejection of a suitable career option, a poorer outcome than a decision made in the absence of social influence. It is therefore important to consider the extent to which copying own-gender or gender-stereotypic models can lead to negative task performance, relative to no social learning.

In the current study, the tendency of both adolescent (Exp 1) and adult (Exp 2) participants to copy male and female models in gender stereotype congruent and incongruent roles is examined, to interrogate this pattern in adolescence and to examine whether it differs in adulthood. A multiple-choice quiz task was developed in which participants were required to answer difficult questions in stereotypically masculine (e.g., plumbing) or feminine (e.g., nursing) domains. For each question, there was one incorrect answer presented as chosen by a male model and one presented as chosen by a female model; the correct answer was never paired with a model. In both experiments, it was predicted that participants would copy the model answers, and that this social learning would negatively affect accuracy. It was also expected that model choice would be affected by stereotype congruence, with more female models copied in female-stereotypic domains, and males copied in male-stereotypic domains. Gender stereotype endorsement (as measured by the GA scale [7]) was expected to be higher in males, and positively associated with the tendency to make gender-stereotypic model choices.

## Experiment 1: Gender biases in adolescent social learning

Copying biases have been extensively studied in children [23, 37, 38] and adulthood [for review see 39], but little research has examined social learning biases in adolescence. This is somewhat surprising given the important life decisions that are made at this time, and the distinctive developmental pattern of social influences in adolescence. There is a gradual change from the dominant influence of family relationships in early childhood to peers by mid-adolescence, such that peers are a key influence in teenage decision-making [40–43]. Accordingly, the teenage years are associated with high peer conformity in both attitudes and behaviour. In terms of attitudes toward STEM subjects, Raabe et al. showed that high school students’ preferences were strongly related to those of their close friends, who were overwhelmingly same gender [6]. The strong influence of own-gender peers at this stage, combined with any own-gender bias in competence judgements, may lead adolescent participants to copy own-gender models. On the other hand, cultural gender stereotyping continues to be a strong influence in UK adolescents, and there is a reduction of own-gender bias in favour of cultural stereotypes of competence by this age group [26, 27]. Thus, it seems plausible that teenaged participants’ social learning choices will be more influenced by gender stereotypes than own-gender bias. In the current experiment, it is therefore predicted that model choice will be gender-congruent rather than own-gender biased (i.e., female models will be copied in stereotypically-female domains, and male models in masculine domains).

Following Wood et al. [7], additional features of Exp 1 were to examine the gender-congruence of participants’ real-word decisions by assessing school subject choices, and to use the GA scale to assess gender stereotype endorsement. This allowed an examination of the relationship between participants’ gender stereotype attitudes, gender stereotype bias in social learning, and gender stereotype bias in subject choice.

Exp 1 was preregistered (see https://doi.org/10.17605/OSF.IO/9Y5BS) with the following hypotheses:

1. Participants will use social information (i.e., in the multiple-choice quiz, they will be more likely to pick an answer paired with a model than one without). This reliance on (inaccurate) social information will mean participants select the correct answer less than would be expected by chance.
2. People will copy the model whose gender is congruent with the gender stereotype of the domain.
3. There will be a relationship between a gender copying bias (a score of how many times a participant selectively copies the stereotypically congruent gender), scores on the gender attribute scale (i.e., gender stereotype endorsement), and gender bias in education choices (i.e., tendency to choose stereotypically own-gender subjects).

### Method

#### Participants

Participants were recruited from a secondary school based in Dundee, Scotland, after appropriate permission was sought from the local education authority, the school, and caregivers. Data were collected in November and December 2021, with no identifiable information stored in the dataset. The preregistered recruitment aim was 128 participants, based on a medium effect size (*η*_p_^2^ = .06; power = .8). However, an additional stopping point was included stating that testing would end when all eligible pupils from the participating school who provided consent had completed the study, which reduced size of the final sample. Seventy-eight participants consented, of which five were excluded because they completed fewer than eight of the 16 test questions. Due to the focus on male-female gender differences, a further four participants were excluded for identifying their gender as ‘other’ (*n* = 3) or ‘prefer not to say’ (*n* = 1). One participant did not record their age but was retained in the analysis because their year of study ensured they fell within the requisite age range. The final sample of 69 participants comprised 37 (53.6%) males and 32 (46.4%) females aged 13 to 17 years, with a mean age of 15.1 years (SD = 0.94).

### Design

#### Quiz experiment

The quiz experiment had a within-subjects design with the variable ‘question domain’ (stereotypically masculine or feminine). Participant gender was included as a between-subjects variable (male or female). The key dependent variables were the proportion of participants’ answers that were correct, the proportion that were (incorrectly) copied from a model, and the proportion of copied answers in which the model gender was congruent with the question domain (‘congruence score’).

#### Copying congruence, gender attitude scale, and subject choice

The correlational part of the study explored relationships between congruence scores in the quiz, stereotype endorsement scores in the Gender Attitude scale, and selection of school subjects stereotypically associated with the participant’s own gender.

The research was conducted with the approval of Abertay University’s Research Ethics Committee (EMS4840).

### Materials

The study was hosted on Qualtrics and could be completed online on any smart device using a mouse or touchscreen.

#### Quiz

The quiz was developed for this experiment and built on Qualtrics. Six photos of six different individuals were obtained from the 10k US Adult Faces Database [44] to serve as fabricated previous quiz participants. These photos were of three females and three males of the same race (white), the same age (mid-twenties), and attractiveness (four out of five on a Likert scale). Three individuals of the two genders were chosen, and one male and one female randomly paired and used each time, to avoid other potential extraneous variables influencing model choice (e.g., judgements of attractiveness to trustworthiness) that might occur if just one male and one female were used for all participants. The quiz comprised of 24 questions within eight topics. Four of these topics were stereotypically associated with females (feminine domains included the topics of Art, Performing arts, Languages, and Care) and four were stereotypically associated with males (masculine domains included the topics Sport, Machines, Science, and Computers); these stereotypic associations had been confirmed in a similar sample in Wood et al. [7]. For each topic there was one easy question and two difficult questions. Questions were piloted (*N* = 12) to ensure that easy questions were correctly answered by everyone, and difficult questions were not answered correctly by anyone. Participants were given a short response window of 12 seconds per question to remove the opportunity to research the correct answer (e.g., through an internet search engine). Extended details of male and female photo selection, as well as quiz question selection, can be found in S1 File.

The order of the question domains (e.g., care, sport) was randomised for each participant. The order of the questions within each domain was fixed as Dummy question, Test question 1, then Test question 2. The position of the four answer choices within the onscreen quadrant was randomised for each question. For dummy and example questions, the male and female faces were both displayed in the quadrant of the correct answer, and their horizontal order (i.e., male or female to the left) was randomised. For Test questions 1 and 2 the placement of male and female faces next to an incorrect answer was randomised, with the constraint that the faces were presented with different answers.

#### Gender Attribute scale

The Gender Attribute (GA) scale is a self-report questionnaire measuring knowledge and endorsement of gender stereotypes [7]. The scale was amended from the original published version to equalize the number of masculine and feminine items (by adding the masculine ‘Firefighter’). The final scale comprised 34 items (12 academic subjects and 22 occupations), of which half were stereotypically associated with females (e.g., Art, Drama, dancer, florist) and half with males (e.g., Science, Maths, pilot, engineer, see S1 File). Each item included a question exploring gender stereotype knowledge (“Who do most people think would be better at this subject/occupation?”) and one exploring personal endorsement (“Who do you think would be better at this subject/occupation?”). The four possible answer choices were always ‘boys’ (‘men’ for occupation items), ‘girls’ (‘women’ for occupation items), ‘both’ and ‘don’t know’.

#### Subject choices questionnaire

This was comprised of 16 school subjects (see S1 File). The subjects were taken from the participating school’s subject choices list and categorised as masculine or feminine according to the findings of Wood and colleagues [7]. Eight choices associated with masculine subject stereotypes (e.g., chemistry, computing) and eight choices with feminine subject stereotype associations (e.g., art, drama) were presented. Participants were requested to tick as many of the subjects listed as they had chosen to study in school.

### Procedure

In each participating class, a teacher provided participants with a link to the Qualtrics experiment during class time. Once participants accessed the link, an information sheet and consent form were displayed on the screen, stating that the purpose of the research was to assess general knowledge and societal attitudes. Following the collection of demographic data (age and gender), the participants were presented with a general knowledge quiz, introduced with the following text:

“*In this section we will assess your general knowledge*. *You will now be presented with 24 multiple choice questions on a number of topics*. *This is a challenging quiz and you only have 12 seconds to respond to each question*. *You’ll see a timer counting down from 12 to 0 like the picture below [image of a timer]*. *The questions in each topic get increasingly difficult*, *so don’t worry if you have to guess the answer*! *To help*, *we have provided the responses from two trial participants who agreed for their responses to be shared*. *Here are the two participants whose answers you will see [pair of images*, *one male and one female image]*. *When a quiz question appears*, *you will be given a choice of four possible answers*, *only one of which is correct*. *The previous participants’ responses will be indicated by the image of their face appearing beside their answer*. *Please click on the blue box with an arrow to see an example question*.*”*

**Fig 1:**
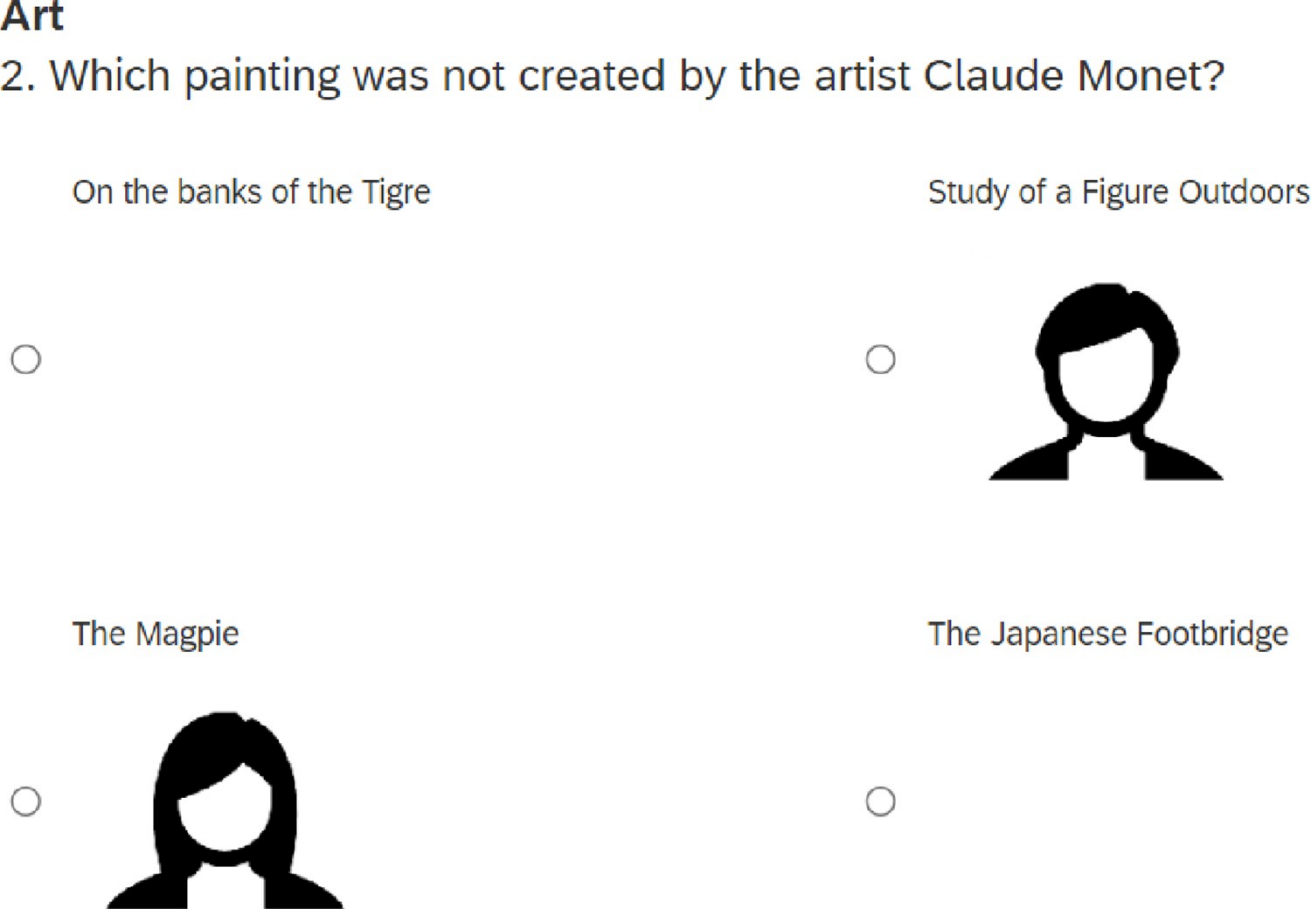
Example question. Example quiz question from the feminine domain (faces shown as icons here were presented as photographic images for participants). For Test questions the correct answer was never paired with a model and the models were always randomly paired with different incorrect responses.

The introduction was followed by one example question. The subsequent general knowledge quiz consisted of 16 questions divided into eight domains (see S1 File for a list of the domains and questions). Within each domain, the first question (Dummy) was designed to be within common knowledge and the remaining two (Test questions 1 and 2) were designed to be difficult. Four answer options were shown in a quadrant below each of the quiz questions and included a picture of a male and a female, presented as previous participants, indicating their own answers (see Fig 1 for an example item). In Dummy questions, both male and female were displayed as having given the correct answer. The purpose of the Dummy questions was to indicate that the models were able to give correct and agreeing answers. For the Test questions, male and female images were always next to two different incorrect answers.

Following the general knowledge quiz, GA scale was introduced with the following text: “*Thank you for completing that challenging general knowledge quiz*. *Next*, *we would like you to think about subjects at school and occupations and what kinds of people might be better at these*. *The following questions have no time limit*. *You will see a school subject or an occupation listed*. *First*, *we want you to tell us who most people would think is better at this*. *Second*, *we want you to tell us who you think is better at this*. *So for each subject there are two questions*. *You can give the same*, *or different answers*, *for each of these two questions*. *Please click on the circle on the left next to the option you agree with most*. *There is no right or wrong answer*, *we just want to know what you think*!*”*

The pairs of the questions were always displayed together in a fixed order of knowledge question followed by endorsement question for the same subject/job. The order of the subject/job pairs was randomised within two separate sections for subjects followed by the section with jobs and the answer choices for each individual question were also displayed in random order. Following completion of the GA scale, participants were presented with a screen showing a list of school subjects offered by their school with the following text: “*Below is a list of subjects offered at [school name]*. *Please select the subjects you have chosen to study this year (if you are in S4*, *S5 or S6)*, *or intend to study next year (if in S3)*. *This could be at National 4*, *National 5*, *Higher or Advanced Higher level*. *Tick as many as apply”*. Following the completion of this section, participants were shown debriefing information which explained the purpose and design of the experiment in detail. There were no post-experimental inquiries.

### Data analysis

#### Data coding, exclusions and manipulation

For the Quiz, participants’ answers were automatically coded by Qualtrics into four response categories: ‘Correct’ (the correct response), Consistent Incorrect (an incorrect response paired with a model whose gender was congruent with the stereotype domain), Inconsistent incorrect (an incorrect response paired with a model whose gender was congruent with the stereotype domain), and Incorrect (an incorrect response not paired with either model).

For both masculine and feminine question domains, counts were performed of the three analysis-relevant response types: (1) correct responses, (2) responses that matched a model answer (i.e., use of social information), and (3) model-matched answers that were stereotype congruent (i.e., gender stereotype congruent copying biases). These count scores contributed to separate analyses to address the experimental hypotheses. Some questions were unanswered due to the 12-second response window, and not all incorrect responses were matched with a model. Therefore, scores were all converted into proportions, relative to the number of questions answered per domain. One exclusion was applied to the data: participants who responded to fewer than 50% of the questions were not included in the analysis, as this response level suggested they were insufficiently engaged in the task.

For the Gender Attribute scale, participants responses to the Gender Attributes Scale were calculated following the procedure of Wood et al. [7]. Specifically, participants’ responses for knowledge and endorsement of stereotypes in masculine and feminine domains were automatically coded by Qualtrics into three response categories: -1 for stereotypical gender incongruent answers, 0 for “both” and “don’t know” options and +1 for stereotypical gender congruent answers. Knowledge and Endorsement scores were calculated for Masculine items (masculine knowledge/endorsement scores, divided by the number of total masculine knowledge/endorsement questions answered) and Feminine Items (feminine knowledge/endorsement scores, divided by the number of total feminine knowledge/endorsement). From here, a rejection score (knowledge minus endorsement scores) was also calculated.

For the subject-choice questionnaire, participant’s responses were categorised by an experimenter as either own-gender congruent or other-gender congruent (see Table S2 in S1 File). Scores of an own-gender subject bias was calculated by dividing the number of own-gender congruent subjects by the total number of masculine and feminine subjects taken at school.

#### Analysis

For the main quiz data, three separate analyses were pre-registered: use of social information (the number of times a participant picked an answer paired with a model, divided by the total number of questions answered by that participant), correct responses (the number of correct answers, divided by the total number of questions answered for each participant), and selection of model copied (the total number of stereotype-congruent models copied, divided by the total number of times a model was copied). This produced continuous data that allowed exploration of response patterns combined across the eight items, in order to examine the effects of gender and gender stereotypes on model copying. All analyses were completed using SPSS Version 28. For the first two analyses, scores were tested against a one-sample proportion of 0.5 (for use of social information) and 0.25 (for correct responses), given that there were four possible choices, and these proportions would indicate ‘chance’ responses (pre-registered analysis). Additionally, exploratory GLMs were conducted to investigate whether participant gender or question domain impacted scores. To test gender stereotype biases in use of social information, scores were tested against a one-sample proportion of 0.5, given that there were two models, and these proportions would indicate ‘chance’ responses The selection of models copied was also investigated using a GLM with question domain (masculine or feminine) and participant gender (male or female) entered as within- and between- subjects factors respectively (pre-registered analysis).

To analyse the relationship between any biases found in model selection and participant’s gender attitudes, Pearson’s correlations were conducted. Pre-registration analysis stipulated that correlational analysis would include the variables: (1) gender stereotype congruent copying bias, (2) gender stereotype endorsement (across both domains), and own-gender subject bias. However, due to the separate patterns for males and females for gender stereotype congruent copying bias in the masculine and feminine domains above, exploratory analysis examined masculine and feminine congruence scores separately.

Data collection and analyses plans for the study were preregistered and are available at https://osf.io/ybtkw/. The Exp 1 dataset can be accessed at https://osf.io/9y5bs.

## Results

### i) Use of social information

Hypothesis 1 predicted that participants would use social information, indicated by the number of times they picked an answer paired with a model compared to an answer without a model. A one sample t-test showed that participants picked an answer paired with a model 74% of the time, significantly greater than would be expected by chance at 50%; *t*(68) = 9.35, *p* < .001, *d* = .21. Hypothesis 1 was therefore supported.

A mixed general linear model was run to explore whether the tendency to copy a model varied by question domain (masculine, feminine) or participant gender (male, female). This analysis revealed no main effects or significant interactions (stereotype domain *F*(1,67) = .00, *p* = .951, *η*_p_^2^ = .00; participant gender *F*(1,67) = .01, *p* = .908, *η*_p_^2^ = .00; stereotype domain x gender *F*(1,67) = .01, *p* = .920, *η*_p_^2^ = .00), suggesting that overall copying levels did not differ between masculine and feminine domains, nor between males and females.

We noted that the reliance on (inaccurate) social information would result in participants selecting the correct answer less often than would be expected by chance (25%). A one sample t-test showed that participants selected the correct answer 12% of the time (Ratio Correct; *M* = .12, *SD* = .13; range = 0 to .50), significantly less than would be expected by chance; *t*(68) = 8.51, *p* < .001, *d* = .13.

### ii) Selection of model copied

While the overall tendency to copy a model did not vary by gender or stereotype domain, it was predicted that these factors would be important in determining *which* model was selected to copy. Hypothesis 2 stated that participants would tend to copy a model whose gender was congruent with the stereotype domain (e.g., copying the male’s answer in a stereotypically masculine question), rather than incongruent (e.g., copying a female’s answer in a stereotypically masculine question).

Congruence was tested against a baseline level of 0.5, whereby 0 would indicate no congruent copying was taking place and 1 would indicate model copying was completely congruent with the domain stereotype (e.g., only copying males in masculine questions). A one sample t-test indicated that when a model was copied, the choice of whether to copy a congruent or incongruent model was not above chance overall (*M* = .50, *SD* = .16; *t*(68) = 0.12, *p* = .908., *d* = .16), so Hypothesis 2 was not supported.

**Fig 2:**
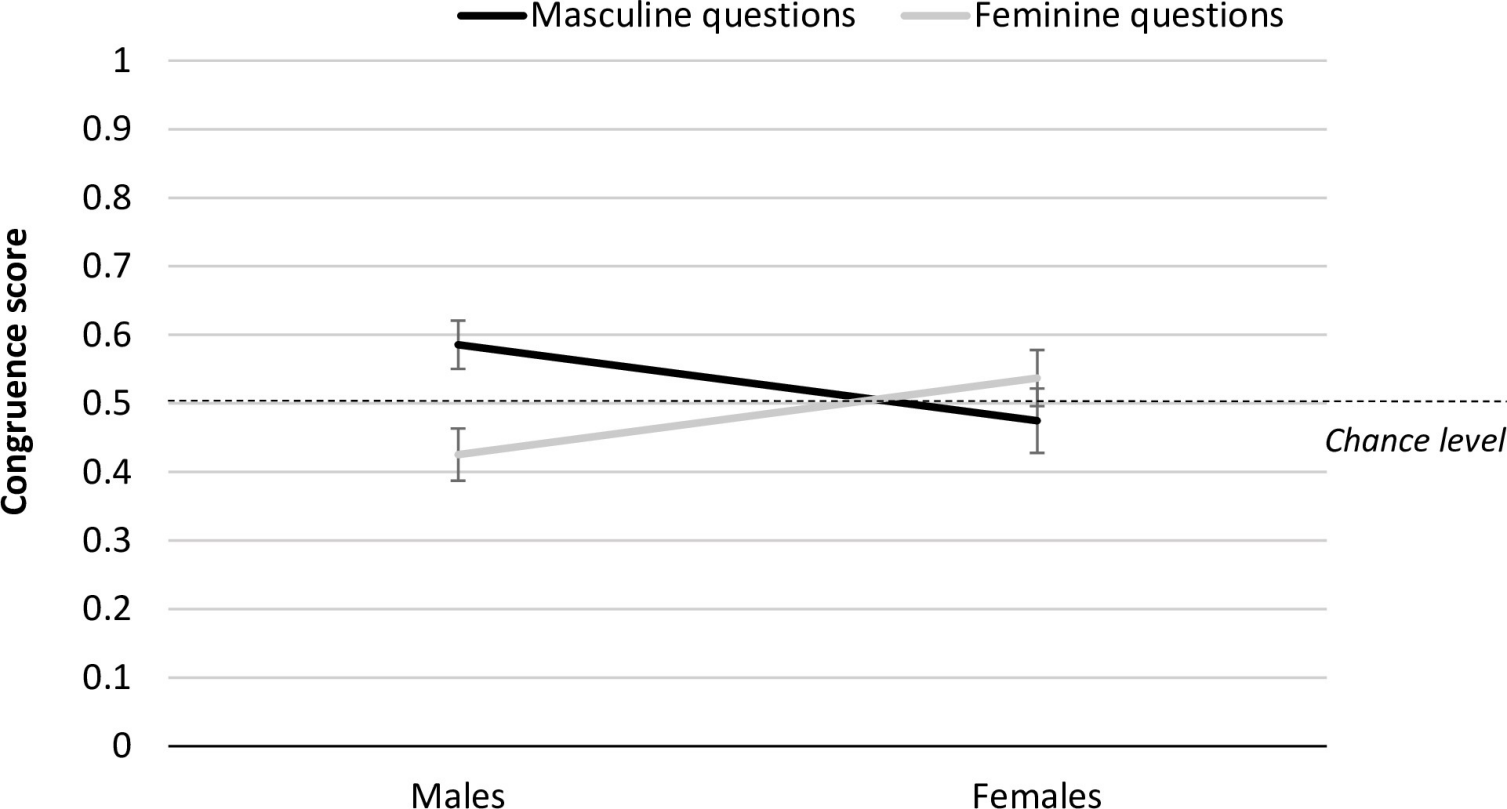
Congruency score in masculine and feminine question domains by male and female participants. Error bars represent one standard SE of the mean.

To explore whether there were different patterns of congruent copying varied by participant gender and stereotype domain, a mixed general linear model was run with congruence score as the DV. This revealed no effect of question domain, *F*(1,67) = 1.39 *p* = .242, *η*_p_^2^ = .02 or participant gender, *F*(1,67) = .00, *p* = .991, *η*_p_^2^ = .00. However, these effects were complicated by a significant stereotype domain X participant gender interaction, *F*(1,67) = 7.17, *p* = .009, *η*_p_^2^ = .10. Follow up paired samples t-tests showed that this interaction was driven by boys copying males regardless of domain (see Fig 2); male participants tended to show congruent copying in the masculine domain (*M* = .59, *SD* = .21), but incongruent copying in the feminine domain (*M* = .43, *SD* = .23; *t*(36) = 3.07, *p* = .004, *d* = .32). In contrast, there was no difference in the congruency score for females in the two domains (feminine domain, *M* = .54, *SD* = .23; masculine domain: *M* = .47, *SD* = .27, *t*(31) = -.94, *p* = .353, *d* = .37).

To determine whether the tendency for boys to copy males within each question domain was reliable, male participants’ congruence scores in the masculine and feminine domains were compared against chance choice of model gender (0.5). It was found that boys copied males more often than chance in the masculine domain, a significant congruence effect (*M* = .59, *SD* = .21, *t*(36) = 2.43 *p* = .02, *d* = .21); the *incongruence* effect in the feminine domain had a similar effect size but did not reach the threshold of significance (*M* = .43, *SD* = .23, *t*(36) = -1.96, *p* = .058, *d* = .23). Overall, boys showed a significant bias to copy an own-gender model, *t*(36) = 3.10, *p* = .004, *d* = .25), whereas girls did not, *t*(31) = .849, *p* = .402, *d* = .27).

### iii) Relationship with gender attitudes

Detailed analysis of gender attitudes is not relevant to the current study, so these data are reported in the S1 File. However, the main findings replicated those reported by Wood and colleagues [7], showing that participants tended to reject stereotypes (i.e., show greater gender stereotype knowledge than endorsement, *F*(1,67) = 140.64, *p* < .001, *η*_p_^2^ = .68), and that this stereotype rejection was significantly higher in girls than boys, *F*(1, 67) = 54.076, *p* < .001, *η*_p_^2^ = .45. Exploratory analysis revealed that in boys, endorsement of stereotypes was higher for masculine items, *M* = .51, *SD* = .31, than feminine items, *M* = .18, *SD* = .47; *t*(36) = 3.43, *p* = .002, *d* = .58). Girls did not show this pattern, with a non-significant difference of means in the opposite direction (masculine *M* = .07, *SD* = .25; feminine *M* = .17, *SD* = .21, *t*(31) = -1.45, *p* = .158, *d* = -37).

Hypothesis 3 predicted there would be a correlation between participants’ gender stereotype endorsement, their copying congruence score (i.e., their tendency to selectively copy the stereotypical gender congruent model), and their own-gender subject choice bias (i.e., their tendency to choose a stereotypical gender congruent school subject). Overall, males’ academic subject choices comprised 72.9% own-gender (masculine) subjects, while females chose 47.4% own-gender (feminine) subjects. Due to the separate patterns of congruence identified in the masculine and feminine domains above, exploratory analysis examined masculine and feminine congruence scores separately.

As Table 1 shows, a significant relationship was found between endorsement of masculine items and own-gender subject bias, *r*(59) = .40, *p* = .002. When broken down by participant gender, the relationship between endorsement of masculine items and own-gender subject bias remained significant for boys, *r*(32) = .494, *p* = .004, but no association was found for girls, *r*(27) = -.108, *p* = .591. The only other relationship to emerge from the correlation analysis was between endorsement of masculine items and congruency score in the masculine domain (i.e., a bias to copy males in masculine questions), *r*(69) = .25, *p* = .036. Interestingly, there was also a tendency for high stereotype endorsement of masculine items to be associated with an incongruence in the feminine domain (i.e., a bias to copy males in feminine questions), although this did not reach significance, *r*(69) = -.22, *p* = .067. These relationships were relatively weak, and not sustained when examined in males and females separately. There was no significant correlation between own-gender subject bias and either masculine (*p* = .705) or feminine (*p* = .351) congruence. This analysis provides partial support for Hypothesis 3, suggesting that endorsement of masculine stereotyped items (but not feminine items) is associated with both an own-gender subject choice bias (especially in boys), and a tendency to copy males when using social information.

**Table 1:** Pearson correlation (r) between scores on the gender attribute scale, and copying congruency score in masculine and feminine domains.

Variable	2	3	4	5
1. Feminine Endorsement	-.09	.07	-.03	.12
2. Masculine Endorsement		-.22	.25*	.40**
3. Feminine congruence			-.12	-.12
4. Masculine congruence				.05
5. Own-gender subject bias				

* *p* < 0.05 (2-tailed)

** *p* < 0.01 (2-tailed)

## Discussion

Experiment 1 was designed to examine adolescent participants’ use of inaccurate social information presented by models, and the extent to which own-gender bias and gender stereotypes would influence the use of this information. It was found that participants did use the social information, copying information provided by male and female models. This had the effect of reducing their accuracy on a difficult general knowledge quiz, where they performed at a below-chance level. Interestingly, gender stereotypes did not seem to drive selection of which model to copy, with participants not choosing stereotype-congruent models above chance level overall. However, model choice did vary by gender: females chose a similar proportion of stereotype-congruent and incongruent models across question domains, while male participants’ model choices were driven by an own-gender bias. Adolescent boys were significantly more likely to copy an answer provided by a male in the masculine questions, and they showed a marginal tendency to also copy males even in the feminine domain. Boys and girls showed higher stereotype knowledge than personal endorsement of stereotypes, indicating stereotype rejection. However, replicating previous research [7], girls showed significantly higher stereotype rejection than boys. Finally, there were positive correlations between gender attitudes and gender-bias in decision-making, both within the quiz (i.e., congruence scores) and the participants’ academic subject choices, but only for stereotypes of masculine items.

Notwithstanding the gender patterns that shaped model choice, the first finding of Exp 1 is that social learning can be detrimental. Even when perceivers have no information about model competence, this suggests that participants tend to gamble on social information being useful in uncertain situations. This finding is consistent with other social influences such as the strength of the informational influence shown in conformity research [45]. Further, both young children and adults show increased copying of others when they are put in an uncertain environment [14, 15], or have not had previous experience with the task [33]. However, in a social learning context that affects real-life decision making, it is important to confirm that model copying can be disadvantageous.

In the real world, the extent to which model copying is likely to be beneficial is dependent on model competence, so it is important to understand the basis for competence judgements. In Exp 1, it was found that perception of model competence was not driven by cultural gender stereotypes as expected. There was no tendency to copy the model whose gender was congruent with the stereotype, despite Gender Attribute scale scores confirming that participants were very aware of cultural stereotypes associating genders with specific academic subjects and occupations. Previous research shows that adolescents tend to replace early own-gender biases with stereotypic beliefs that, say boys are better at maths (and more likely to show brilliance in general) whereas girls are better at languages [7, 27]. However, participants’ awareness of domain specific cultural stereotypes did not drive their model choice in the current experiment. While gender stereotypes were not determinants of social learning choices, participant gender was an important factor. For female participants, the tendency to choose a male or female model when copying an answer was around chance level for both masculine and feminine questions; there was no reliable own-gender or gender-stereotype bias. In contrast, male participants showed significantly different congruence levels in the masculine and feminine domains, with a congruence effect emerging in the masculine questions, and a tendency towards an unexpected incongruence effect in feminine domains. Thus boys showed a significant own-gender bias in their model choice, whereas girls did not.

Despite the clear gender patterns shown in the quiz task, the experiment is limited by the sample size (*N* = 69), which was considerably smaller than the preregistered aim of 128 participants. This issue arose because data was collected in a single school due to practical constraints of testing in the immediate post-pandemic era. The preregistered sample size aim was based on power analysis with a medium effect size, as the task was novel and the size of the predicted gender-congruence effect therefore difficult to predict. While the null finding regarding the gender congruence effect therefore has to be interpreted with caution, there is no evidence to suggest a tendency in the predicted direction, with the congruence main effect *p*-value above .9. However, the key finding that an own-gender bias unexpectedly dominated social cognition in male participants was reliable even within the relatively small size of the current sample.

The own-gender bias in male participants’ model choice was echoed in their academic subject choices. Replicating previous research [7], it was found that boys’ choices corresponded to gender stereotypes more than girls’; masculine subjects comprised nearly three-quarters of male choices, while females chose approximately half masculine and half feminine subjects from the available list. It should be acknowledged that a limitation of the measure of subject choice in the current experiment is that in order to provide an equal number of masculine and feminine subjects to choose from, only a sub-set of 16 subjects were presented. This prevented a bias in the quantity of masculine and feminine subjects available for participants to select within the experiment but may have created an alternative bias by omission (e.g., more popular masculine than feminine subjects being included in the list). While this potential bias cannot be excluded, the stereotypic subject choices are consistent with the more stereotypic attitudes of boys overall. While both boys and girls showed significant rejection of cultural stereotypes (i.e., higher stereotype knowledge than personal endorsement), the level of rejection was higher in the girls, again replicating the finding from a similar population reported by Wood and colleagues [7].

An additional element of the stereotype endorsement pattern was that boys had stronger stereotypes of own-gender items, relative to their endorsement of female stereotypic items. There was no evidence of this pattern in girls. This suggests that for the male participants only, the stereotype that males are better suited to masculine subjects and careers remains robust. The correlational analysis revealed that endorsement of masculine stereotypes was significantly positively associated with both own-gender subject bias and a tendency to copy male models in masculine questions. These patterns demonstrate that participants’ stereotypic beliefs about suitable roles for males were associated with decisions made both in the social learning task and in the real world. When examined within boys and girls separately, the only relationship that remained significant was in male participants, between endorsement of masculine items and own-gender subject bias. The finding that only in boys was there a strong correlation between stereotypic attitudes and bias to own-gender subjects replicates previous findings obtained in a larger sample [7] and highlights the potential influence of masculine stereotypes in boys’ decision-making.

Overall, the gender differences in subject choice and model choice found in Exp 1, along with the relative strength of the masculine stereotype, suggests that adolescent boys may be more inclined to conform to masculine expectations of subject and career choices, than girls to conform to female expectations. Further, the social learning findings suggest that these patterns may be linked to a tendency for boys to copy other boys. An important question is whether these patterns are specific to current adolescents, or whether the same gender difference in own-gender biases are prevalent in adults. Experiment 2 was designed to address this question.

## Experiment 2: Gender biases in adult social learning

Gender biases in social learning in adults have not received extensive empirical attention to date, with most studies focusing on early childhood social cognition. From a cognitive perspective it could be argued that post-adolescence, executive functioning improvement should lead to a reduction in reliance on stereotypes [46]. However, adult cognition remains widely influenced by the stereotypic associations that are acquired across development [47] and the prevailing gender imbalance in careers may lead to more working hours in adulthood being spent in environments dominated by one gender, than school hours in adolescence [8]. Cultural stereotypes of gender competence are therefore likely to remain influential, potentially leading to more reliance on gender stereotypes in uncertain situations relative to the pattern shown by adolescent participants in Exp 1. Further, the reduced levels of peer conformity shown in adulthood [42] and increase in stable romantic relationships that are majority opposite-gender [48] means that an own-gender bias may be less prominent in adult social cognition. It seems likely that social learning will therefore be more influenced by cultural gender stereotypes than an own-gender bias.

To explore these predictions, Exp 2 followed the method of Exp 1 in an adult population across a broad age range, with two amendments to the Exp 1 design: school subject choice questions were omitted as they were not applicable to this older sample, and age was included as an exploratory covariate because a wide age range was targeted in recruitment. This age range allowed us to examine changes in copying behaviour across adulthood rather than just in the narrow gap between adolescence and early adulthood, with the potential for cohort effects and differences in experience to influence patterns of reliance on models as well as developmental changes. The hypotheses from Exp 1 were repeated in Exp 2 (see https://doi.org/10.17605/OSF.IO/A23UV), so it was predicted that:

1. People will use social information (i.e., be more likely to pick an answer paired with a model than one without). This reliance on (inaccurate) social information will mean participants select the correct answer less than would be expected by chance.
2. People will copy the model whose gender is congruent with the gender stereotype of the domain.
3. There will be a correlation between a gender copying bias (a score of how many times a participant selectively copies the stereotypically congruent gender) and scores on the gender attribute scale.

## Method

### Participants

In total, 305 participants were recruited online via a university participation scheme and in the local community via convenience sampling. Data were collected in January and February 2022, with no identifiable information stored in the dataset. Forty participants were excluded due to answering fewer than eight of the 16 quiz questions. The final sample (*N* = 265) consisted of 96 males and 169 females aged 18 to 82 years (*M* = 36.05 years, *SD* = 16.1), with no difference in age between males and females, *t*(263) = .785. *p* = .447. This sample is comfortably above the recommended minimum of 128 for the factorial design (see Exp 1).

### Design, materials, and procedure

The design and materials were identical to Exp 1 except that the school subject choice questionnaire was not included. The procedure was also the same except that the study was accessed via an online Qualtrics link and was completed individually by participants in their own time rather than in a school setting. The research was conducted with the written approval of Abertay University’s Research Ethics Committee (EMS5017) and the University of Aberdeen School of Psychology Ethics Committee (PEC/4791/2021/9). There were no post-experimental inquiries.

### Data analysis

Data coding, exclusions, and manipulation was identical to that of Exp 1 except that there was no subject-choice questionnaire. The preregistered analysis was also identical to Exp 1 except that age was entered as a covariate within GLMs. The Exp 2 dataset can be accessed at https://osf.io/9y5bs.

## Results

### i) Use of social information

Hypothesis 1 stated participants would use social information, indicated by the number of times participants picked an answer paired with a model compared to an answer without a model. A proportion model copying score was calculated as in Exp 1, and a one sample t-test showed that participants picked an answer paired with a model 65% of the time, significantly greater than would be expected by chance at 50% (*t*(264) = 11.18, *p* < .001, *d* = .22), supporting Hypothesis 1.

**Fig 3:**
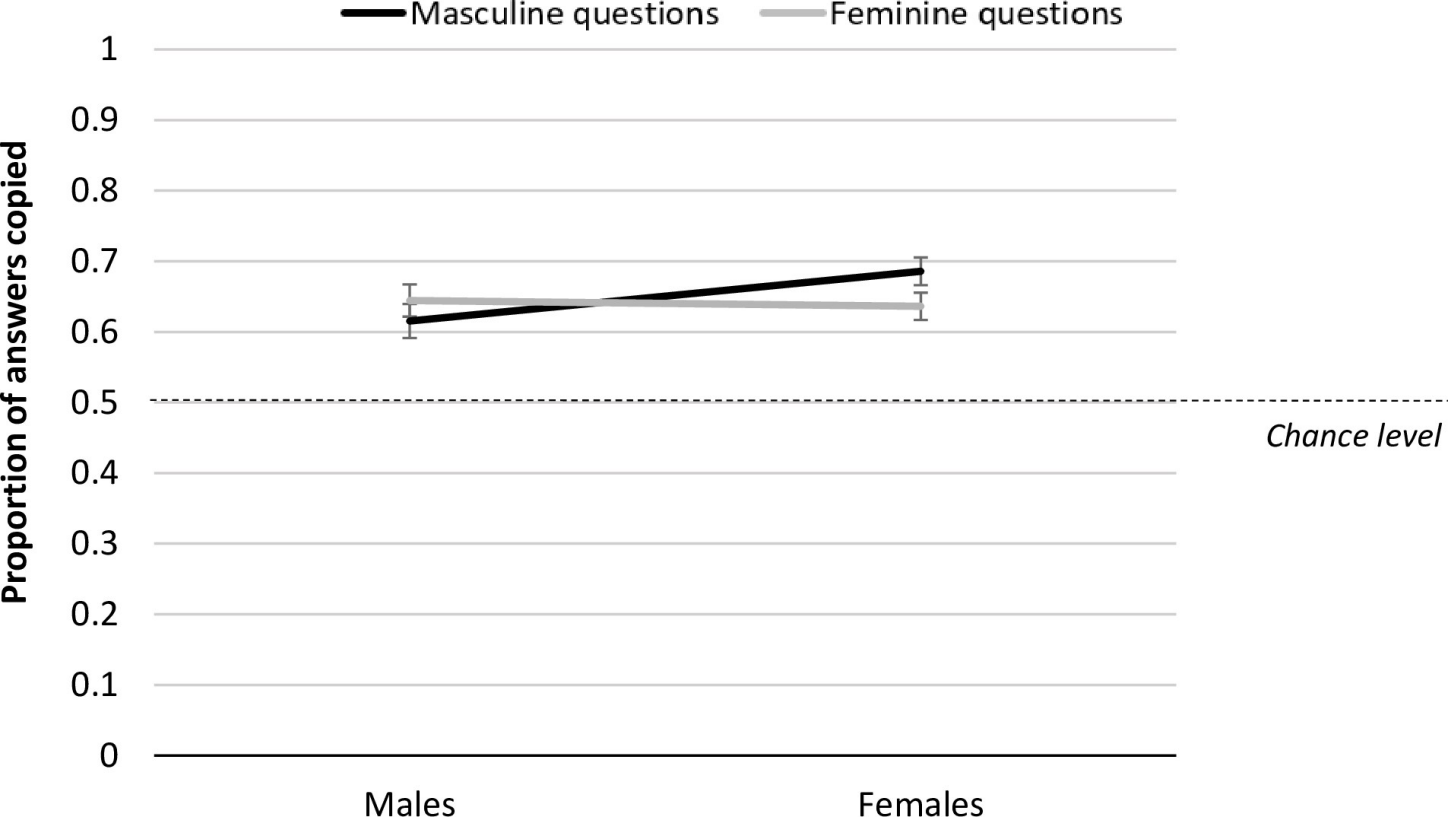
Proportion of answers copied from model answers by question domain and participant gender. Note. Error bars represent one standard SE of the mean.

A mixed general linear model was run to examine whether overall copying varied by question domain (masculine, feminine) and participant gender (male, female), with age in years included a covariate. Age was significantly negatively related to the tendency to copy a model, *F*(1,262) = 18.561, *p* < .001, *η*_p_^2^ *=* .07; *r*(265) = -.253, *p* < .001. There was no main effect of question domain *F*(1,262) = .817, *p* = .367, *η*_p_^2^ = .00 or participant gender *F*(1,262) = .820, *p* = .366, *η*_p_^2^ = .00, but there was a significant interaction between these factors, *F*(1,262) = 6.942, *p* = .009, *η*_p_^2^ = .03 after controlling for age. Female participants copied answers paired with a model significantly more in masculine (*M* = .68, *SD* = .26) than feminine questions (*M* = .64, *SD* = .25; *t*(168) = 2.78, *p* = .006, *d* = .23), while male participants showed no reliable difference in copying between the two domains, *t*(95) = 1.25, *p* = .215, *d* = .23. Thus female participants only showed a significant tendency to rely on social information more when the question was associated with the opposite gender (see Fig 3).

As in Exp 1, we noted that reliance on social information would result in participants selecting the correct answer less often than would be expected by chance (25%). A proportionate correct answer score was calculated as in Exp 1, and a one sample t-test showed that participants selected the correct answer 16% of the time (Ratio Correct; *M* = .16, *SD* = .12; range = 0 to .53), significantly less than would be expected by chance, *t*(264) = 11.86, *p* < .001, *d* = .12.

### ii) Selection of model copied

Hypothesis 2 predicted participants would copy a model whose gender was congruent with the question domain. As in Exp 1, the effects of gender and stereotype domain on the selection of which model to copy were examined by calculating a proportionate congruence score, with a score of 0 indicating no congruent copying and 1 indicating complete congruency. A one sample t-test against chance level congruence (0.5) indicated a significant congruence effect (*M* = .53, *SD* = .19), *t*(264) = 2.54, *p* = .012, *d* = .19, supporting Hypothesis 2.

To explore whether the tendency to copy congruent models varied by gender and stereotype domain, a mixed general linear model was run with congruence score as the DV and age in years included as a covariate. There was positive covariance between age and congruence score but no significant linear relationship, *F*(1,262) = 4.409, *p* = .037, *η*_p_^2^ = .02; *r*(265) = -.096, *p* = .118. No main effects or interactions were found for congruence scores after controlling for age: question domain, *F*(1,262) = .313, *p* = .576, *η*_p_^2^ = .00; participant gender, *F*(1,262) = .093, *p* = .760, *η*_p_^2^ = .00; question domain x gender *F*(1,262) = 1.795, *p* = .181, *np2* = .01.

### iii) Relationship with gender attitudes

Participants’ scores on the GA scale were calculated as in Exp 1. Full exploration of these scores is included in the S1 File but in brief, as expected participants showed stereotype rejection with higher knowledge than endorsement scores, *F*(1,250) = 529.68, *p* < .001, *η*_p_^2^ = .68, and females showed higher stereotype rejection than males, *F*(1, 250) = 11.10, *p <* .001, *η*_p_^2^ = .04. Exploratory analysis revealed that in adult males, endorsement of stereotypes was higher for masculine items, *M* = 0.28, *SD* = 0.29, than feminine items, *M* = 0.23, *SD* = 0.24; *t*(86) = 2.41, *p* = .018, *d* = .20). As with adolescents, adult females did not show this pattern (masculine *M* = 0.15, *SD* = 0.19, feminine *M* = 0.17, *SD* = 0.19, *t*(164) = -1.12, *p* = .267, *d* = .14).

Hypothesis 3 predicted there would be a correlation between participants’ gender stereotype endorsement and their copying congruence score (i.e., their tendency to selectively copy the stereotypical gender congruent model). To be consistent with the Exp 1 analysis, correlations were explored in the masculine and feminine domain separately (see Table 2). The masculine and feminine endorsement scores were highly correlated with one another, *r*(252) = .722, *p* < .001, a relationship that remained significant when male and female samples were examined separately (males: *r*(87) = .720, *p* < .001; females: *r*(165) = .715, *p* < .001). No other significant relationships emerged, with only a weak negative association between masculine stereotype endorsement and congruency in the feminine domain approaching significance, *r*(252) = -.120, *p* = .057.

**Table 2:** Pearson correlation (r) between scores on the gender attribute scale, and copying congruency score in masculine and feminine domains.

Variable	2	3	4
1. Feminine Endorsement	.72***	-.04	-.04
2. Masculine Endorsement		-.12	.02
3. Feminine congruence			.03
4. Masculine congruence			

*** *p* < 0.001 (2-tailed)

## Discussion

In Exp 2, the purpose was to determine whether the Exp 1 findings were limited to an adolescent sample for whom peer influence is strong, or whether adults show the same reliance on social influence and sources of bias in model choice. Replicating the pattern found in adolescents in Exp 1, it was found that adult participants did use social information in the form of models’ answers, and that this negatively affected their performance on the general knowledge quiz. Interestingly, the tendency to rely on social information reduced with age across the sample of 18- to 82-year-olds, matching a tendency for quiz performance in Exp 2 to be slightly higher than that of Exp 1 (16% v. 12%, both still significantly below chance). The current experimental design does not allow us to determine the cause of the age-related change in model copying. It may reflect a developmental change in participants’ knowledge, and confidence to apply that knowledge even when others disagree, or a general tendency to attend less to social learning cues across the lifespan. Alternatively, there could be important cohort differences across the sample, reflecting diverse cultural histories of participants at different current ages. Exploring these explanations would be a valuable topic for future research.

Copying choice varied by question domain in female participants, who showed significantly more copying in the masculine than feminine domain (controlling for age). In contrast, male participants did not show a reliable pattern of copying more in one domain. This pattern suggests adult female participants tend to rely more on social information when the domain does not match the area of expertise stereotypically associated with their own gender, perhaps indicating internalisation of own-gender negative stereotypes in women [49]. In terms of which model was copied, participants were more likely to select a stereotype-congruent than incongruent model and, in contrast to the pattern found in adolescent participants, this effect did not vary by gender. Again, age was included as a covariate in the analysis but did not significantly moderate the effects of gender or domain. This pattern suggests that both men and women are more likely to copy females in a stereotypically feminine domain, and to copy males in a stereotypically male domain, following the competence association of cultural gender-stereotypes rather than an own-gender bias.

Analysis of participants’ scores on the GA scale suggested a similar pattern to the adolescent sample in Exp 1, with a tendency to reject the cultural stereotypes (i.e., to show higher stereotype knowledge than personal endorsement scores). Again, females showed higher rejection than males and males alone had a pattern of higher endorsement of masculine items than feminine items. Gender attitudes were not significantly related to gendered copying however, which is perhaps surprising given that the overall congruence effects suggested model choice was underpinned by stereotypic associations. It may be that implicit attitudes would be more reliably predictive of stereotype congruence in social learning, as the social influence is unlikely to be conscious [50–52; although see 53]. Overall, adult copying was in a stereotypic direction, but not directly related to expressed stereotype endorsement.

## General discussion

Across two experiments, the effects of gender stereotypes and own-gender bias on model choice were tested, in a difficult multiple choice quiz task. Across both age groups, participants tended to use social learning in their quiz responses, preferring to copy answers shown as being chosen by models than those previously unchosen, negatively affecting task performance. In adults, the tendency to copy model answers reduced with age, but did not moderate the pattern of model choice. The patterns of competence judgements varied across experiments, suggesting either a developmental or cohort shift. In the adolescent sample (Exp. 1), there was no evidence of gender stereotypes of competence driving model choice (e.g., copying females when the question was in a stereotypically feminine domain), but there was a tendency in the male participants for male models to be copied regardless of domain. This own-gender bias was not apparent in the female participants. In the adult sample (Exp 2), there was no evidence of an own-gender bias even in male participants, but copying choice was affected by cultural gender stereotypes: both men and women were more likely to copy a male model in masculine questions and a female model in feminine questions. Female participants also showed a tendency to rely more on social learning for male questions than those in the female domain, a pattern that was not present in the adolescent sample.

The age differences in social influence in male participants was particularly striking. In Exp 1, adolescent boys showed an unexpected own-gender bias, tending to copy male model answers regardless of domain. However, the adult males tested in Exp 2 did not show this pattern, showing instead a gender-stereotype bias in their tendency to copy a model whose gender was congruent with the domain. The adult male pattern was predicted on the basis of gender-stereotypic competence beliefs which remain culturally prevalent and show increases with age for both men and women [54, 55]. Model choice is known to be influenced by competence beliefs, especially in tasks in which the correct behaviour choice is unclear [5]. The distinctive adolescent own-gender pattern may also be competence-based, reflecting a genuine belief in boys that boys know better than girls (e.g., the brilliance stereotype [26, 56]). In the real world, the significant pressure of (predominantly same-gender) peer influence that peaks in adolescence may combine with an own-gender brilliance belief in boys to drive a social learning bias to copy other boys.

Age differences in female participants’ copying choices across Exp’s 1 and 2 showed a different pattern, with no evidence of a strong own-gender or gender-stereotypic biases in adolescence, but the predicted gender-stereotype bias emerging in adulthood. Rather than a developmental increase in stereotype reliance, it is possible that this pattern reflects a cohort effect, with the younger generation of girls perhaps more educated against gender bias. Although this is highly speculative, there was a tendency for adult (but not adolescent) females to copy a model more when the question was in a stereotypically masculine domain, suggesting internalisation of gender competence stereotypes in women [49]. This finding suggests that more could be done to increase adult women’s perception of female competence in traditionally masculine domains.

In addition to examining overall gender patterns, inclusion of the Gender Attribute scale [7] provided additional insight into the individual differences in stereotyping that might underpin gender biases in social influence. GA scale scores showed that across both experiments, there was strong stereotype rejection, and this was consistently higher in females than males. As Wood and colleagues suggest [7], this pattern is likely to reflect females’ greater awareness of the relative costs of gender stereotypes to their own group. In Exp 1, adolescents showed no significant association between endorsement of masculine and feminine stereotypes. Although this null finding should be treated with caution due to the relatively small sample, there was no evidence that adolescents who endorse male stereotypes also endorse female stereotypes. In contrast, the adults in Exp 2 showed a high correlation between endorsement of masculine and feminine stereotypes, suggesting gender stereotypes are less independent of one another in these adults. At this stage, gender attitudes may be reflective of more general attitudes toward gender roles (i.e., egalitarian vs. traditional), a developmental shift from a high own-gender stereotype awareness. Own-gender awareness may be particularly prominent in adolescence, the post-puberty period in which body consciousness is high and associated with objectification by others [57, 58]. Alternatively, this pattern could again be a cohort effect with the older generations’ gender attitudes being interlinked in a way that is not part of the culture of younger people. As well as this age pattern, an important gender difference emerged in both experiments with endorsement of male stereotypic items being significantly higher than female items in adolescent boys (Exp 1) and men (Exp 2), a pattern that was not found in girls or women. It would appear that females’ endorsement of gender roles has been effectively challenged; in contrast, the stereotypic perception in males, particularly for male competence, remains strong.

The link between these attitudes and other aspects of decision-making is important. In Exp 1, male endorsement of masculine stereotypes was associated with selecting more own-gender academic subjects at school. Similarly, boys who had high endorsement of masculine stereotypes showed stronger social influence in the quiz answers. These data suggest that in adolescence, endorsement of male stereotypes (i.e., stereotypes of male competence) is predictive of own-gender bias in decision-making. In adulthood however, there was no robust relationship between masculine stereotype endorsement and model choice. Overall, adults tended to show more reliance on congruent models regardless of the level of stereotype endorsement they expressed.

While patterns of association cannot provide information on causal relationships, a consistent emergent pattern across the current measures and experiments is that addressing gender biases in decision making is likely to involve tackling stereotypes of male competence, especially in boys and men. Currently, counter-stereotypic interventions tend to be targeted toward females, challenging the stereotype that males are more suited than females for leadership and STEM roles [59]. The current findings suggest that this is not enough to tackle subject and career gender imbalances. It is males whose attitudes are most stereotypic, and this may have implications for women too–if men consider male subjects and occupations unsuitable for women and girls, this can create a challenging ‘chilly climate’ that thwarts female inclusion [60–62]. The lower endorsement of female stereotypic items somewhat contradicts previous research suggesting that competence associations for female academic abilities (i.e., in verbal domains) tend to be higher than in male domains (i.e., science and maths [27, 28, 63]). This difference may be a local cultural artefact but highlights the need to explore these patterns separately in male and female participants, to clarify patterns that may be obscured when data is combined.

The clear message from both the model choice data and patterns of gender stereotype endorsement in the current study is that if gender imbalances are to be addressed, particularly in young people, males need to be targeted by interventions. Interventions should not focus only on attracting males into the industries in which they are under-represented such as healthcare and education [31], but on challenging their own-gender stereotypes (e.g., in STEM), which may currently exclude girls and women. Further, the current findings argue against basing interventions on opposite gender examples and role models, given adolescent boys’ own gender bias in social learning. Using information on competence biases in social influence could be a valuable strategy for influencing decision making, and while more research is needed to replicate and extend the current findings, the tendency for teenage boys to copy boys is consistent with research showing own-gender peer influence from a wide network in boys [64], and provides and important starting point for this research base.

In terms of social influence, the current findings also make an important point about the issue of negative effects of model-based biases in social learning. In the current study the task used was affected by the informational influence, with difficult questions inducing conformity with apparent previous participant answers. The task design meant that these model answers were always incorrect, meaning that social learning genitively impacted performance. While this may not be a pattern typically found in the type of social learning associated with cultural evolution (e.g., puzzle boxes [5, 16]), it is important to acknowledge in terms of socially learned information that might be counter-productive, such as stereotype reinforcement or risk-taking behaviours. Further, own gender biases in adolescence and gender-stereotypic biases in adulthood may lead to ineffective model choice due to inaccurate perceptions of competence [36]. The costs of social influence are worthy of further investigation.

There are a number of limitations of the current study that could also be addressed by future research. In particular, the small sample size and limited geographical spread of participants in Exp 1 means that the findings should be replicated in larger and more diverse cultural groups, where variability in family background, exposure to stereotypic cultural norms and experience of educational interventions may impact results [65, 66]. A goal for future research would also be to examine data from transgender and non-binary individuals. Here, no distinction between cisgender and transgender participants was recorded and there were insufficient numbers of non-binary individuals to allow meaningful analysis. However, it seems likely that the attitudes and experiences of individuals who are transgender or do not identify as male or female will differ in important ways from a cisgender male and female sample, potentially impacting patterns of gender stereotype and own-gender biases.

An additional limitation of Exp 1 is the potential bias in subjects available for selection to indicate own subject choices, which may not capture the full array of all individuals’ school choices. For both experiments, the use of a difficult multiple-choice quiz was adopted in order to increase chances of social influence, so that gender patterns could then be observed, however from a social learning perspective, an interesting follow-up would be to explore the extent to which the patterns observed here were elicited by easier questions, in which participants are more likely to have relevant extant knowledge to weigh against that of the available models. Further, participants implicit attitudes were not tested; it is possible that additional relationships between stereotypic attitudes and social influences would be revealed if attitude information were collected at an implicit level. Finally, differences in social influence between the samples tested in Exp 1 and Exp 2 suggest that there may be developmental influences to examine more closely, using a longitudinal design. In particular, the clear shift from an own-gender to a gender-stereotypic bias in the male participants needs further research, to elucidate the developmental trajectory from childhood to adulthood.

In conclusion, the current study provides evidence for the first time that decision-making in both adolescence and adulthood are impacted by gender biases. Adolescent males show an own-gender bias, a tendency to copy other boys, while adult males and females tend to show a cultural gender-stereotypic bias, a tendency to copy a model whose gender matches the stereotypic domain of the question. While gender stereotype endorsement was lower than knowledge of cultural stereotypes overall, male participants showed a particular failure to reject stereotypes associated with masculinity. Endorsement of masculine stereotypes was associated with both academic subject choice and a tendency to copy boys in adolescence, and a reluctance to copy female models in feminine domains in adults. More work needs to be done to challenge gender stereotype endorsement, particularly in adolescent boys, if persistent gender biases in education and the workplace are to be addressed.

## Supporting information

S1 File Additional methodology information and data analyses for Experiments 1 and 2. (DOCX)
