# Supplementary material for: The cost of social influence: Own-gender and gender-stereotype social learning biases in adolescents and adults
Source: PLoS One. 2023 Aug 11;18(8):e0290122. doi: 10.1371/journal.pone.0290122 (PMC10420340; doi:10.1371/journal.pone.0290122)
Supplement: S1 File — (DOCX) [file pone.0290122.s001.docx]

**Supplementary materials**

**i) Male and female photo selection**

Six photos of faces (three male, three female) were selected in four stages from 2,222 photos taken from 10k US Adult Faces Database, previously rated on demographic attributes including age, ethnicity, fame status and attractiveness by 30 participants [44].

In the first stage of selection, photos that appeared to be taken outdoors, from a yearbook or in a professional photography studio were excluded in order to give credibility to the experimental set up that the photos were of previous participants, taken by the researchers in a laboratory environment. In the second stage of selection, only forward-facing photos of non-famous individuals with white ethnicity (to match the majority of the target demographic) were selected. In the third stage, faces were selected that matched the target demographic majority age range (average age rating between 1.5 and 2.5 on a five-point Likert scale, where a rating of two represents 20 to 30 years of age), and were of slightly above average attractiveness (average attractiveness rating between 3.5 and 4.5 on a five-point Likert scale).

Finally, two experimenters agreed on the final six photos from the selected set. All faces were smiling. All males had short hair and all females had past shoulder-length hair and natural/no make-up. These can be identified within the 10k US Adult Faces Database by (fabricated and not necessarily gender congruent) names: ‘Albert_Waring_1_oval’, ‘Google_1_Howard Lindsey_10_oval’, ‘Google_1_Janet Lippert_1_oval’, ‘Google_1_Mark Shilling_7_oval’, ‘Google_1_Philip Patton_1_oval’, and ‘Google_1_Thomas Mccormick_19_oval’.

Photos were marginally adapted to match for lighting and shape (oval).

**ii) Gender Attribute Scale**

An example of an item can be seen in Figure S1, while the full list of items is presented in Table S1.

**Figure S1: Example question from the of Gender Attributes scale**


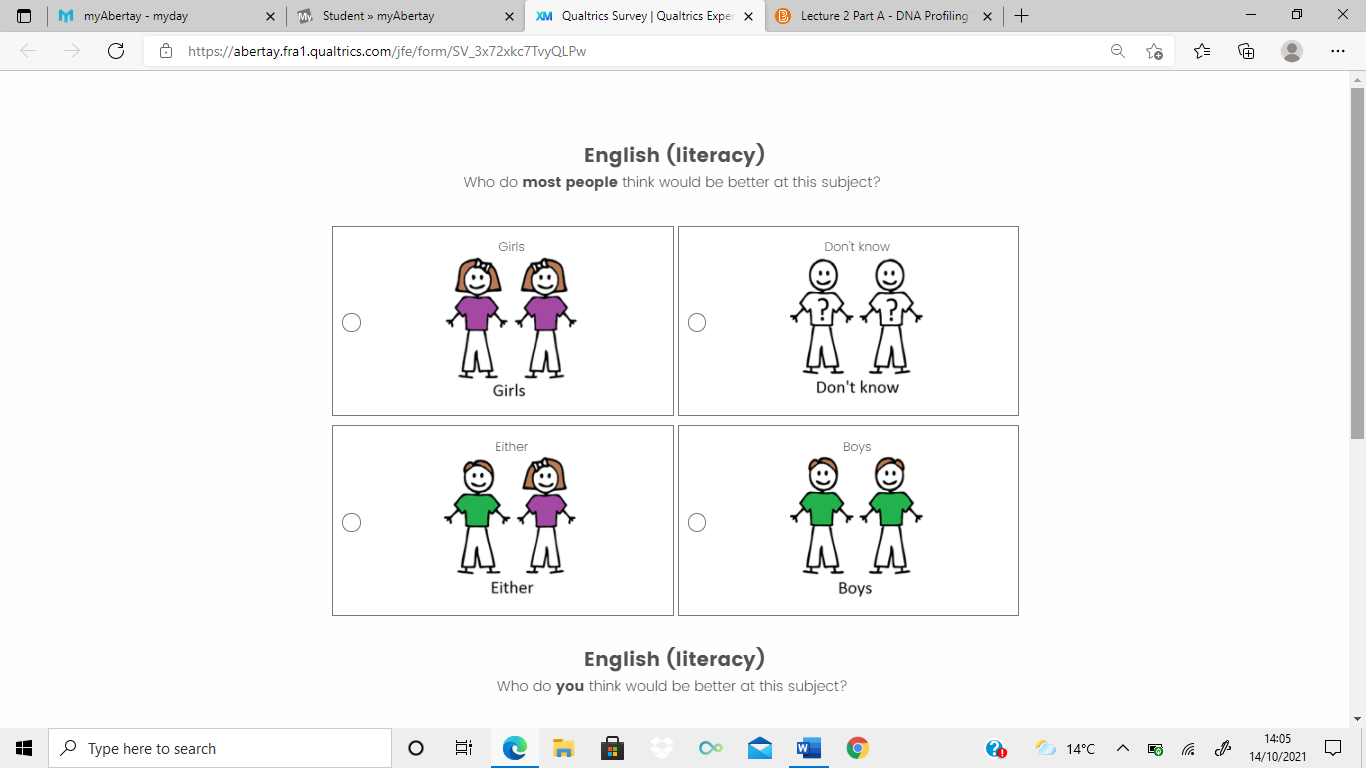


**Table S1 Gender Attitude sale items**

| **Masculine items** | **Feminine items** |
| --- | --- |
| Maths (Numeracy | English Literacy |
| Design and Technology | Music |
| Sport (PE) | Drama |
| Science | Art |
| IT (Computing) | Modern Languages |
| Geography | Stay at home parent |
| History | Telephonist |
| Scientist | Teacher |
| Police Officer | Shop Assistant |
| Mechanic | Secretary |
| Plumber | Nurse |
| Pilot | Cleaner |
| Lorry Driver | Hairdresser |
| Firefighter | Florist |
| Builder | Dressmaker |
| Farmer | Paid childcare |
| Engineer | Dancer |

**iii) School choices**

The list of sixteen school subject choices is shown in Table S2.

**Table S2: School subject choices**

| **Masculine subjects** | **Feminine subjects** |
| --- | --- |
| PE | French |
| Engineering Science | Art/photography |
| Graphic Communication | Music |
| Computing | Drama |
| Chemistry | Spanish |
| Physics | German |
| Biology/ human biology | Early education and childcare |
| Technical subjects (e.g. Design and Manufacture, Practical Woodwork, Practical Metalwork) | Home Economics (e.g., Health and Food, Practical Cookery, Fashion and Textile) |

**iv) Quiz question selection**

Using data from Wood and colleagues [7], eight topics of questions type were identified to produce four stereotypically associated with females (henceforth ‘feminine domain’) and four stereotypically associated with males (‘masculine domain’, see Table S3). Within each topic, the researchers created easy questions that were assumed to be correctly answered by most British adolescents and adults, and difficult questions that were assumed to be too difficult for most British individuals of any age. The questions were paired with four responses: one correct and three incorrect.

The list of questions was piloted with 12 people to select the final question set. ‘Easy’ questions were excluded if any pilot participant did not know the correct answer. ‘Difficult’ questions were excluded if any pilot participant expressed confidence about the correct answer from the selection given. From the remaining list, questions were selected based on roughly equal word length and diversity of topic. The final list of questions and responses included in the study is shown in Table S3.

**Table S3: List of questions and possible answers. The first answer shown in the table is the correct answer.**

**Masculine domain:**

| ***Topic*** | ***Easy “dummy” question*** | ***Difficult test question i*** | ***Difficult test question ii*** |
| --- | --- | --- | --- |
| Sport | **Who won the 2012 Olympic Tennis Singles Men's Gold medal?**  Andy Murray  Jamie Murray  Andy Robertson  Chris Hoy | **In archery, what is a fistmele?**  Brace height  Type of bowstring  A part that absorbs the sideways force of an arrow  Hand position when drawing a string | **Which prime number is the number of teams that played in the 1st Football World Cup?**  13  19  11  17 |
| Proportion Correct | | 0.07 | 0.16 |
| Machines | **The logo of which car brand comprises four interlocking circles?**  Audi  Toyota  Jeep  Fiat | **Which of the following is not included in the list of pre-flight aeroplane essential checks?**  Lontiors  Ailerons  Props  Fuel contagion | **In plumbing, what is checked during MaP testing?**  Bulk removal  Valve seals  Water flow  Pipe strength |
| Proportion Correct | | 0.22 | 0.05 |
| Science | **Which of these gases do we need to breathe?**  Oxygen  Helium  Methane  Krypton | **Which of the following is a notable type of nuclear reaction:**  Spallation  Thermation  Flectation  Munation | **In the Grand Unified Theory of particle physics, what form can 16 fermions take?**  Octonion  Serfactum  Pluntron  Farbium |
| Proportion Correct | | 0.04 | 0.2 |
| Computers | **What does RAM stand for?**  Random access memory  Read Actual Memory  Register all memory  Remote access Machine | **When was the first 1GB disk drive released?**  1980  1985  1983  1987 | **Which amongst the following is not an advantage of Distributed systems?**  Reliability  Incremental growth  Resource sharing  None of the above |
| Proportion Correct | | 0.12 | 0.13 |

**Feminine domain:**

| ***Topic*** | ***Easy “dummy” question*** | ***Difficult test question i*** | ***Difficult test question ii*** |
| --- | --- | --- | --- |
| Art | **What is the name of Britain's famous graffiti artist?**  Banksy  Bansko  Banky  Bank | **Which painting was not created by the artist Claude Monet?**  On the banks of the Tigre  The Magpie  Study of a Figure Outdoors  The Japanese Footbridge | **Who won the 2020 Muse Fashion Design Award for their runway collection?**  Traces  Blending Soul  Uncertain Certainty  Shan Shui |
| Proportion Correct | | 0.13 | 0.11 |
| Performing Arts | **Which actor played the leading role in 'A Star is Born'?**  Lady Gaga  Saoirse Ronan  Jennifer Hudson  Carey Mulligan | **Which of these choreographers is not associated with postmodern dance?**  Jean Coralli  Martha Graham  Isadora Duncan  Merce Cunningham | **Which theatre is on Broadway?**  Majestic Theatre  Dominion Theatre  Fortune Theatre  Novello Theatre |
| Proportion Correct | | 0.17 | 0.16 |
| Languages | **Vert is what colour in French?**  Green  Red  Blue  Orange | **What does the word ‘boffola’ mean?**  A joke  A tramp  An attack  A chant | **Which historical novel was written by Colleen McCullough?**  The Thorn Birds  Three Day Road  The Historian  Silence |
| Proportion Correct | | 0.29 | 0.30 |
| Care | **Which of these is a classic ingredient in pesto?**  Basil  Oregano  Mint  Thyme | **What educational approach is associated with Dimitrios Thanasoulas?**  Constructivist learning  Problem-based learning  Self-directed learning  Competency-based learning | **What type of dressing is used to treat a skin burn?**  Jalonet  Silicone  Calcium alginate  Adherus |
| Proportion Correct | | 0.15 | 0.08 |

**v) Adolescent participants’ GA scale scores (Exp 1)**

Participants’ proportionate stereotype knowledge and endorsement scores were submitted to a 2 (Participant gender: male v. female) x 2 (Stereotype level: knowledge v. endorsement) x 2 (Domain: masculine v. feminine) mixed ANOVA. This revealed no main effect of Gender *F*(1,67) = 0.724, *p* = .398, *η*_p_^2^ = .011, but a significant main effect of stereotype level *F*(1,67) = 140.64, *p* < .001, *η*_p_^2^ = .68, complicated by a Gender x Stereotype level interaction, *F*(1,67) = 54.076, *p* < .001, *η*_p_^2^ = .44. As can be seen in Figure S2, stereotype knowledge was higher than stereotype endorsement overall, but this pattern was stronger in girls (*p* < .001) than boys (*p* =.020). Calculating a rejection score (knowledge minus endorsement) confirmed that stereotype rejection was significantly higher in female (*M* = .48, *SD* = .24) than male (*M* = .11, *SD* = .17) participants, *t*(67) = 7.354, *p* < .001.

**Figure S2: Knowledge and endorsement scores in male and female adolescents (Exp 1)**

The only other significant finding to emerge from the ANOVA was a Gender x Domain interaction, *F*(1,67) = 12.593, *p* < .001, *η*_p_^2^ = 16. This arose because male adolescents (but not females) showed significantly higher stereotyping of masculine than feminine items (*p* < .001). There was a tendency for female adolescents to show higher stereotyping scores on feminine than masculine items, but this did not reach significance (*p* = .062; see Figure S3).

**Figure S3: Masculine and feminine stereotypes in male and female adolescents (Exp 1)**

**vi) Adult participants’ GA scale scores (Exp 2)**

Participants’ proportionate stereotype knowledge and endorsement scores were submitted to a 2 (Participant gender: male v. female) x 2 (Stereotype level: knowledge v. endorsement) x 2 (Domain: masculine v. feminine) mixed ANOVA. As in Exp 1, this revealed no main effect of Gender *F*(1,250) = 2.610, *p* = .107, *η*_p_^2^ = .010, but a significant effect of stereotype level *F*(1,250) = 529.680, *p* < .001, *η*_p_^2^ = .679, complicated by a Gender x Stereotype level interaction, *F*(1,250) = 11.100, *p* < .001, *η*_p_^2^ = .043. As can be seen in Figure S4, stereotype knowledge was higher than stereotype endorsement overall; while this difference was significant in both males and females (both *p* < ,001), the difference was slightly larger in females. As in Exp 1, a rejection score was calculated (knowledge minus endorsement) and this confirmed that stereotype rejection was significantly higher in female (*M* = .46, *SD* = .28) than male (*M* = .37, *SD* = .30) participants, *t*(250) = 2.533, *p* = .012, *d* = .29.

**Figure S4: Knowledge and endorsement scores in males and female (Exp 2)**

The ANOVA also revealed a main effect of Domain, *F*(1,250) = 12.540, *p* < .001, *η*_p_^2^ = .048, with higher stereotyping (across knowledge and endorsement) of masculine than feminine items. However, as can be seen in Figure S5 there was a significant interaction between Domain and Gender, *F*(1,250) = 5.342, *p* = .022, *η*_p_^2^ = .021, with males giving significantly higher scores for the masculine than feminine items (*p* <.001) while the females’ scores did not differ significantly across domains (*p* = .296).

**Figure S5: Masculine and feminine stereotypes in males and females (Exp 2)**

Finally, there was a significant three-way interaction between Gender, Domain and Stereotype level, *F*(1,250) = 4.309, *p* = .039, *η*_p_^2^ = .017. Rejection scores were used to explore this interaction, being submitted to a 2 (Participant gender: male v. female) x 2 (Domain: masculine v. feminine items) mixed ANOVA. This transformed the three-way interaction reported above into a Domain x Gender interaction. As Figure S6 shows, this arose because there was significantly higher rejection of masculine than feminine items by female participants (*p* = .003), whereas rejection of masculine and feminine items was similar in male participants (*p* = .677).

**Figure S6: Rejection of masculine and feminine stereotypes in males and females (Exp 2)**
